# Supplementary material for: Women’s autonomy, neonatal, infant and under-five mortality in the Upper East Region of Ghana
Source: PLOS Glob Public Health. 2024 Sep 19;4(9):e0002776. doi: 10.1371/journal.pgph.0002776 (PMC11412492; doi:10.1371/journal.pgph.0002776)
Supplement: S1 Text — (DOCX) [file pgph.0002776.s001.docx]

**Description of Components of the Women’s Autonomy Variables**

|  |  |  |  |  |  |
| --- | --- | --- | --- | --- | --- |
| Variable | Obs | Mean | Std. dev. | Min | Max |
| hhpurch | 17,005 | 3.035107 | 2.376694 | 1 | 9 |
| spenddecider | 13,672 | 1.497586 | 0.8451941 | 1 | 4 |
| dailypurch~s | 13,672 | 1.502048 | 0.8504579 | 1 | 4 |
| freetovisit | 13,672 | 1.657621 | 0.4745229 | 1 | 2 |
| refusesex | 13,672 | 1.308075 | 0.4617146 | 1 | 2 |
| vaccperm | 15,213 | 1.323999 | 0.4680151 | 1 | 2 |
| permission | 13,672 | 1.269529 | 0.4437313 | 1 | 2 |
