# Supplementary material for: Women’s autonomy, neonatal, infant and under-five mortality in the Upper East Region of Ghana
Source: PLOS Glob Public Health. 2024 Sep 19;4(9):e0002776. doi: 10.1371/journal.pgph.0002776 (PMC11412492; doi:10.1371/journal.pgph.0002776)
Supplement: S3 Text — (PDF) [file pgph.0002776.s003.pdf]

In case of reply the number and date of this <u>letter should be quoted.</u>

My Ref: Approg/GEHIP 03/2012
Your Ref:

Your Health. Our Concern

Navrongo Health Research Centre
Institutional Review Board
Ghana Health Service
P. O. Box 114
Navrongo, Ghana
Tel/Fax: +233-3821-22348

Email: irb@navrongo.mimcom.org

19th March 2012

Dr. Frank Kwodjo Nyonator
Project Director/Director PPME
Ghana Health Service
Accra

ETHICS APPROVAL ID: NHRCIRB127

Dear Dr. Nyonator,

Approval of progress report for the protocol titled: *"<u>The Ghana Essential Health Intervention Programme (GEHIP) OF The Tanzania Ghana Health Partnership (TGHP)</u>"*

I write to inform you that the annual progress report for the above named study was reviewed and approved by the NHRCIRB.

Please note that any amendment to the study protocol must receive prior NHRCIRB approval before implementation.

You are also to note that this approval expires on 18th March 2013.

The Board would like to draw your attention to the fact that, the Directorship of the Navrongo Health Research Centre has changed. The new Director is in the person of Dr. Abraham Rexford Oduro. All future correspondence in this respect should therefore carry this current information until you are advised otherwise.

Should you require a renewal of your approval, a progress report should be submitted two (2) months before the expiration date for review.

The Board wishes you all the best in this study.

Sincerely,

Dr. Nana Akosua Ansah
(Interim Vice Chair, NHRC IRB)

Cc: The Director, NHRC
